# Effects of Hierarchical Roost Removal on Northern Long-Eared Bat (*Myotis septentrionalis*) Maternity Colonies

**DOI:** 10.1371/journal.pone.0116356

**Published:** 2015-01-22

**Authors:** Alexander Silvis, W. Mark Ford, Eric R. Britzke

**Affiliations:** 1 Department of Fish and Wildlife Conservation, Virginia Polytechnic Institute and State University, Blacksburg, Virginia, United States of America; 2 US Geological Survey, Virginia Cooperative Fish and Wildlife Research Unit, Blacksburg, Virginia, United States of America; 3 US Army Engineer Research and Development Center, Environmental Laboratory, Vicksburg, Mississippi, United States of America; CSIRO, AUSTRALIA

## Abstract

Forest roosting bats use a variety of ephemeral roosts such as snags and declining live trees. Although conservation of summer maternity habitat is considered critical for forest-roosting bats, bat response to roost loss still is poorly understood. To address this, we monitored 3 northern long-eared bat (*Myotis septentrionalis*) maternity colonies on Fort Knox Military Reservation, Kentucky, USA, before and after targeted roost removal during the dormant season when bats were hibernating in caves. We used 2 treatments: removal of a single highly used (primary) roost and removal of 24% of less used (secondary) roosts, and an un-manipulated control. Neither treatment altered the number of roosts used by individual bats, but secondary roost removal doubled the distances moved between sequentially used roosts. However, overall space use by and location of colonies was similar pre- and post-treatment. Patterns of roost use before and after removal treatments also were similar but bats maintained closer social connections after our treatments. Roost height, diameter at breast height, percent canopy openness, and roost species composition were similar pre- and post-treatment. We detected differences in the distribution of roosts among decay stages and crown classes pre- and post-roost removal, but this may have been a result of temperature differences between treatment years. Our results suggest that loss of a primary roost or ≤ 20% of secondary roosts in the dormant season may not cause northern long-eared bats to abandon roosting areas or substantially alter some roosting behaviors in the following active season when tree-roosts are used. Critically, tolerance limits to roost loss may be dependent upon local forest conditions, and continued research on this topic will be necessary for conservation of the northern long-eared bat across its range.

## Introduction

Roosts provide bats with sites for day-time sheltering as protection from weather and predators, mating, and social interaction. For species in temperate areas that form maternity groups in forested landscapes, roosts also provide thermal benefits for successful juvenile development [1–4]. Because of their importance in both survival and recruitment, roosts long have been considered a critical habitat feature for bats [5, 6]. Approximately half of all known bat species use plants as roosts [6]; in North America, roosts most commonly are found in snags or live trees with cavities or defects. Roosts such as snags in forests are ephemeral [7, 8]. Ephemerality of the roost resource strongly suggests that bats experience roost loss at some low constant background level, with periodic pulses of increased roost loss after intense disturbances from fire, wind throw, ice damage, insect outbreak, or certain types of forest management actions [9–12]. It seems likely, therefore, that bats are adaptive to roost loss. This plasticity often is ignored as many managers tasked with bat conservation often view roosts and roosting areas as fixed landscape elements that are decoupled from stochastic environmental processes [13, 14].

Bat conservation in forested landscapes often involves identification of roost sites with subsequent limitations on management activities (e.g., forestry) within these areas. Conservative approaches to roost habitat management may seem warranted, but this strategy may interrupt natural processes or anthropogenic management actions that are vital to create suitable roosts in the present or provide roosts in the future. Impacts of management actions that result in roost loss are unknown as few studies directly have assessed the effect of roost loss on bat roosting behavior in controlled, manipulative studies. Evidence from roost exclusion studies suggests that exclusion from permanent structures can decrease site fidelity, alter home range size, lower reproductive recruitment, and reduce colony size and the strength of association among individuals [15–18]. Conversely, several lines of evidence suggest that tree roosting bats may be tolerant of roost loss up to some threshold point. For example, bats have exhibited positive roosting responses to prescribed fire at short-term and long-term temporal scales [19–23]. Positive responses to prescribed fire may be due to rapid, increased snag recruitment that offsets the loss of existing snags [24–26]. Clearly, natural forest disturbance processes also can remove and create bat roosts. Natural forest disturbance processes contrast with many types of forest harvest that remove potential and available roosts without creating new roosts in the short-term. However, if applied on the landscape properly, it is possible that forest harvesting may mimic natural processes that also create suitable roosting areas or possibly enhance the quality of existing roosts, i.e., reduce canopy shading of remaining boles.

Tolerance limits to roost loss are unclear and probably highly variable among bat species and the forest systems wherein they reside [15–18, 27, 28]. For colonial species, insight into the impacts of roost loss will require understanding both of individual and colony level factors [29]. Responses to roost loss may be apparent in demographics, survival, roost use, space use, and sociality. Unfortunately, demographic changes are exceedingly difficult to ascertain for bats that roost-switch frequently and exhibit fission-fusion behavior. Within the context of roost use, resilience to roost loss generally may be visible as either a shift in overall uses of individual roosts without a change in overall space use or social structure, or alternatively, as a shift in roosting area and roosts without a change in social structure. Conversely, if colonies are not robust to disturbance, the colony may either dissolve such that social structure at the site is not maintained, or dissolve to the point where no bats are present on the site [27]. Within the network of roosts used by colonies of bats, individual roosts frequently are used differentially, with some receiving intense use (primary roosts) and others limited use (secondary roosts) [29–31]. Roost switching studies have provided insight on why bats may switch roosts, but the underlying causes for differences in the relative level of roost use have not been investigated widely. Regardless, differential roost use suggests that individual roosts may either serve different functions for colonies and individual bats therein or vary in their value. If so, loss of heavily used or primary roosts may impact colonies more strongly than loss of less frequently used roosts [28, 29].

Our objective was to experimentally examine how hierarchical loss of roosts affects roosting social structure along with roost and space use by female northern long-eared bats (*Myotis septentrionalis*) during the maternity season at both the colony and individual level. Northern long-eared bats occur in forests throughout the eastern United States and southern Canada [32–38], but foraging activity consistently is greatest in closed-canopy forests [34, 39–44]. During the maternity season (May-July), female northern long-eared bats form non-random assorting colonies in upland forests under the exfoliating bark or within cavities of snags or declining live trees [10, 33, 36, 44]. This species is a proposed for listing as endangered and currently of high conservation concern in North America (*Federal Register* § 78:61045–61080) due to severe population declines following the onset and spread of White-nose Syndrome in eastern North America. An improved understanding of the effects of roost loss on this species will be important for development of future conservation efforts.

Accordingly, we evaluated the impacts of primary and multiple secondary roost loss specifically to reflect discussion in the literature by Rhodes et al. [29] and Silvis et al. [27] that suggests that loss of either a single primary of >20% of total roosts might result in colony fragmentation, a negative conservation outcome of substantial concern. We assessed changes in colony roost and space use, roost selection, and social structure, as well as changes in individual behaviors related to roost switching. We specified several *a priori* hypotheses related to the differing levels of roost site disturbance based on previous research on multiple species [15, 16, 18, 27, 29]. For primary roost tree removal, we proposed 2 hypotheses:

1. H_1_: At the colony level, loss of the primary roost will result in an alternate tree receiving increased use, subsequently causing a previously less-used roost to become the primary roost [15, 16]; bats will not display evidence of roost seeking behavior. Bats will display an affinity for the same roosting area, but the core use area would re-center around the new primary roost, and roost selection would be consistent. At the individual level, loss of the primary roost will not impact roost switching behavior or distances moved between sequentially used roosts.
2. H_2_: At the colony level, loss of the primary roost will result in dissolution of the colony [29]. Space use will either be random across the former roosting area or will be nonexistent. Bats will display characteristics of roost searching, and the characteristics of selected roosts will differ [18]. At the individual level, loss of the primary roost will increase roost switching frequency and the distances moved between sequentially used roosts.

For secondary roost loss, we proposed three hypotheses:

1. H_1_: At the colony level, loss of multiple secondary roosts will not impact roosting behavior, social structure, space use, or roost selection by northern long-eared bat maternity colonies [27]. At the individual level, loss of multiple secondary roosts will not impact roost switching behavior or distances moved between sequentially used roosts. Roost characteristics will not differ.
2. H_2_: At the colony level, loss of multiple secondary roosts will result in dissolution of the colony [27]. Space use will either be random across the former roosting area or will be nonexistent. Bats will display characteristics of roost searching and roost characteristics will differ [18]. At the individual level, loss of multiple secondary roosts will increase roost switching frequency and the distances moved between sequentially used roosts.
3. H_3_: At the colony level, loss of multiple secondary roosts will result in increased social cohesion and increased use of the primary roost, and roosting area will decrease. Roost characteristics will not differ. At the individual level, loss of multiple secondary roosts will decrease the number of roosts used by individual bats and the distances moved between roosts.

## Methods

We conducted our study at 3 sites on the Fort Knox military reservation in Meade, Bullitt, and Hardin Counties, Kentucky, USA (37.9°N, −85.9°E, WGS84). Our sites lie in the Western Pennyroyal subregion of the Mississippian portion of the Interior Low Plateau physiographic province of the upper South and lower Midwest portion of the USA [45]. Forest cover is predominantly a western mixed-mesophytic association [46], with second- and third-growth forests dominated by white oak (*Quercus alba*), black oak (*Q. velutina*), chinkapin oak (*Q. muehlenbergii*), shagbark hickory (*Carya ovata*), yellow poplar (*Liriodendron tulipifera*), white ash (*Fraxinus americana*), and American beech *(Fagus grandifolia*) in the overstory, and sassafras (*Sassafras albidum*), redbud (*Cercis canadensis*), and sugar maple (*Acer saccharum*) in the understory [47].

We initially captured northern long-eared bats over small woodland pools from May through July 2011 (pre-roost removal) and 2012 (post-roost removal). We attached a radiotransmitter (LB-2, 0.31 g: Holohil Systems Ltd., Woodlawn, ON, Canada) between the scapulae of each female bat using Perma-Type surgical cement (Perma-Type Company Inc., Plainville, CT, USA). A uniquely numbered lipped band was attached to the forearm of all captured bats. After identifying a small number of roosts, we maximized number of bats captured by erecting mist nets around roosts located while radiotracking bats. Captured bats were released within 30 minutes of capture at the net site. Using TRX-1000S receivers and folding 3-element Yagi antennas (Wildlife Materials Inc., Carbondale, IL, USA), we attempted to locate radio-tagged bats daily for the life of the transmitter or until the unit dropped from the bat. For each located roost, we recorded tree species, diameter at breast height (dbh; cm), height (m), canopy openness (%), decay class ([48]; live [1], declining [2], recent dead [3], loose bark [4], no bark [4], broken top [6], broken bole [7]) and crown class ([49]; i.e., suppressed [S], intermediate [I], codominant [CO], dominant [D]). We estimated size of individual colonies by performing 5 exit counts per colony at day-roosts used by radiotracked bats.

We followed the methods of Silvis *et al.* [27] in defining a northern long-eared bat maternity colony as all female and juvenile bats connected by coincident roost use. We represented colonies graphically and analytically as two-mode networks that consisted of bats and roosts (hereafter “roost network”) [30, 31]. We used these roost network representations to describe patterns of roost use by colonies and to identify roosts for our removal treatments. To reduce bias resulting from uneven tracking periods and observing only a portion of each colony, we considered relationships to be binary (i.e., presence or absence of a connection) [50]. We assessed roost network structure using mean degree, network degree centralization, network density, and clustering. Within networks, degree is a count of the number of edges incident with a node [51]; high degree values indicate a large number of connections to a node. Network degree centralization, density, and clustering all have values between 0 and 1 (0 = low, 1 = high). Network degree centralization describes the extent that a network is structured around individual nodes, whereas network density and clustering describe the distribution of connections among nodes [52–56]. We calculated two-mode degree centralization and density using the methods of Borgatti and Everett [52] and clustering using the method of Opsahl [57] for our roost network. To determine whether our observed network values differed from those of random networks, we performed 999 Monte Carlo simulations and compared observed network metrics to random network metrics using two-tailed permutation tests [58, 59]; random networks [60] were generated with the same number of nodes as our observed networks and with a constant probability of link establishment. We then compared the relative difference from random networks pre-post treatment to assess whether colony social dynamics and roost use patterns were disrupted.

In February 2012 when bats were hibernating and not occupants of trees and snags, we implemented two roost removal treatments and one control following the identification and delineation of 3 colonies in 2011. For our primary roost removal treatment, we felled the single roost with the highest degree centralization value via chainsaw. For the secondary roost removal treatment, we similarly felled 5 randomly selected roosts (24% of colony total) with degree centralization values less than the colony maximum, but greater than the colony minimum in our secondary roost removal treatment group. This number was selected to specifically test the simulation-based predictions of Silvis *et al.* [27] that colonies may fragment with loss of >20% of roosts.

We used conditional Wilcoxon 2-sample tests and conditional Chi-squared tests to compare continuous (height, dbh, and canopy openness) and categorical roost characteristics (species composition, decay stage, and crown class) pre- and post-treatment and among groups; we corrected for multiple comparisons using the Bonferroni method. Conditional tests were performed using Monte Carlo simulations with 999 permutations. We examined the roost switching behavior of individual bats by creating a Poisson regression model describing the number of roosts used by a bat relative to the total number of relocations, reproductive condition, and interaction of treatment identity and year. We used this Poisson model to conduct general linear hypothesis tests with Tukey’s adjustment for multiple comparisons to determine whether the number of roosts used by bats differed within or among treatment areas. We evaluated the fit of our Poisson model using maximum-adjusted D^2^ [61]. We assessed the spatial component of roost switching behavior by individual bats by comparing the distances that bats within treatment areas moved between sequentially used roosts with general linear hypothesis tests, also with Tukey’s adjustment for multiple comparisons. We performed our general linear hypothesis tests for distances moved on a linear mixed model containing year, group, their interaction term, and reproductive condition as fixed effects, and bat identity as a random effect; we used a log transformation to normalize distance data. We assessed the fit of our linear mixed model using the conditional (R^2c^) and marginal (R^2m^) coefficients of determination [62].

We evaluated roost removal impacts on colony roosting area space use for each treatment group using Bhattacharya’s affinity (BA) [63] and the difference in roosting area centroids between years. The BA uses the joint distribution of 2 utilization distributions to quantify similarity between utilization distributions and is appropriate for comparisons of utilization distributions for the same individual or group [63]. These values range from 0 to 1, with values close to 1 indicating highly similar utilization distributions [63]. We calculated 95% utilization distributions from the pooled locations of all bats within a colony using bivariate normal fixed kernel methodology. To reflect the concentration of roost use, we weighted roost locations by the number of times a roost was used by radio-tagged bats [64]. We used the reference method for smoothing parameter estimation as appropriate for weighted locations [65]; that also allowed us to consider our estimates of colony space use as liberal. In cases where roosting areas of separate colonies overlapped to an appreciable extent, we calculated the utilization distribution overlap index (UDOI) to determine if space use was independent; UDOI values range from 0 to infinity, with values <1 indicating independent space use, and values >1 indicating non-independence [63].

We assessed overall changes in colony roost use patterns by comparing pre- and post-roost removal network degree centralization, density, and clustering for the roost networks. We used this same comparative network approach to assess changes in colony roosting social structure for the single mode projections of our 2-mode roost networks [66]. This projection allowed us to focus on existing direct and indirect connections among bats in a colony. Because comparing values from networks of differing size may yield inappropriate inferences [67], we used indirect comparisons of network characteristics. In these, we compared the relative difference between a roost or social network and its equivalent random network pre- and post-treatment. All analyses were performed in the R statistical program version 3.0.2 [68]. We calculated conditional tests using the *coin* package [69], linear mixed models using *lme4* [70], and utilization distributions, BA, and UDOI values using the *adehabitatHR* package [71]. We used the *igraph* [72] and *tnet* libraries [57] to visualize networks and calculate metrics. Lastly, network Monte Carlo simulations were performed using a custom script with dependencies on the *igraph* and *tnet* libraries. We used an α = 0.05 for all tests of statistical significance.

### Ethics statement

Our study was carried out in accordance with state requirements for capture and handling of wildlife (Kentucky Department of Fish and Wildlife Resources permit numbers SC1111108 and SC1311170) and did not involve any endangered species at the time of the study. Capture and handling protocol followed the guidelines of the American Society of Mammalogists [73] and was approved by the Virginia Polytechnic Institute and State University Institutional Animal Care and Use Committee (protocol number 11–040-FIW). We received explicit permission to conduct work on the Fort Knox military reservation from the reservation staff biologists and Fort Knox Range Control. Data used in this study are archived in the Virginia Polytechnic Institute and State University VTechWorks institutional repository (DOI: 10.7294/W4H41PBH).

### Results

We captured 58 female northern long-eared bats pre-treatment in 2011. Based on patterns of coincident roost use, we assigned 36 of these bats (11 gestating, 20 lactating, 1 post-lactation, and 4 non-reproductive) to 3 colonies. Exit counts for these 3 colonies generated minimum estimated colony sizes of 13, 18, and 14 bats, respectively. We captured 67 bats post-treatment in 2012, 62 of which (4 gestating, 45 lactating, 10 post-lactation, and 3 non-reproductive) we were able to assign to the 3 colonies identified in 2011. We recaptured only 3 individuals banded in 2011 during 2012. Exit counts indicated that the 2012 colonies contained a minimum of 24, 20 and 25 bats, respectively. We located 58 roosts over 204 relocation events for the 3 colonies identified in 2011 and 100 roosts (7 of which were used in 2011) over 324 relocation events in 2012. We recorded a mean (± SD) of 5.7 (± 1.5) locations per bat in 2011 and 5.2 (± 2.9) in 2012.

We identified between 4 and 33 roosts per colony pre-roost removal, and between 23 and 42 roosts per colony post-removal (Table 1). When controlling for the total number of relocations of an individual bat and reproductive condition, the number of roosts used by individual bats was similar between pre- and post-treatment and among colonies, with the exception of the control colony, pre-removal, that differed from all other groups (model D^2^ = 0.74; Tables 1, 2).

**Table 1:** Summary of female northern long-eared bat roost use patterns.

	**Control**		**Primary Roost Removal**		**Secondary Roost Removal**	
	**Pre**	**Post**	**Pre**	**Post**	**Pre**	**Post**
Total Roosts Used	4	23	33	42	21	35
Total Relocations	88	86	75	130	41	108
Mean Roosts Used Per Bat	1.2 (± 0.6) ^a,b,c,d,e^	4.4 (± 1.9) ^a^	4.8 (± 1.9) ^b^	3.6 (± 2.0) ^c^	4.1 (± 1.6) ^d^	3.2 (± 1.8) ^e^
Median Non-Zero Roost Switching Distance	111.1 (± 157.6)	147.6 (± 180.1)	156.2 (± 103.2)	161.9 (± 114.4)	100.4 (± 146.7) ^a^	219.4 (± 173.8) ^a^
Kernel Density 95% Roosting Area (ha)	1.3	58.3	50.0	32.3	45.3	41.1
Bhattacharya’s Affinity	NA	0.12	NA	0.75	NA	0.77
Difference in Roosting Area Centroid (m)	NA	258.7	NA	71.2	NA	128.7
Network Degree Centralization	0.99 (>)	0.43 (>)	0.44 (>)	0.72 (>)	0.3	0.28 (>)
Network Clustering Coefficient	0.00	0.69	0.57	0.80 (>)	0.57	0.70 (>)
Network Density	0.30	0.19	0.14	0.08	0.19	0.09

Roosting movement and space use summary metrics for 3 northern long-eared bat (*Myotis septentrionalis*) maternity colonies subjected to different levels of roost removal on the Fort Knox military reservation, Kentucky, USA, pre- and post-roost removal (2011 and 2012) treatment. Where applicable, values are presented with standard deviation (± SD) and significant differences (*P* < 0.05) between groups are indicated by superscripts a–e. Network metrics were calculated directly from the two-mode network consisting of bats and roosts; arrows indicate the direction of difference when metrics differ from random networks.

**Table 2:** Factors influencing the number of roosts used by individual female northern long-eared bats.

**Predictor**	**Parameter Estimate**	**SE**	**z value**	***P*-value**
Intercept	-0.65	0.28	-2.348	0.02
Locations	0.15	0.02	6.442	< 0.001
Post-removal	1.13	0.28	4.018	< 0.001
Treatment: Primary	1.33	0.32	4.486	< 0.001
Treatment: Secondary	1.44	0.28	4.816	< 0.001
Repro: Non-reproductive	-0.26	0.31	-0.843	0.40
Repro: Post-lactation	0.05	0.19	0.255	0.80
Repro: Gestating	-0.14	0.20	-0.711	0.18
Post-removal x Primary	-1.54	0.36	-4.241	< 0.001
Post-removal x Secondary	-1.38	0.33	-4.223	< 0.001

Parameter summary of the Poisson model describing the number of roosts used by female *Myotis septentrionalis* from 3 maternity colonies subjected to different levels of roost removal (2011 and 2012) on the Fort Knox military reservation, Kentucky, USA, pre- and post- roost removal treatment. Locations = number of days bat was located; Repro = bat reproductive condition.

Neither roost dbh nor height differed between treatments or among colonies (Table 3). Canopy openness was similar between pre- and post-treatment, but some individual colonies differed from one another (Table 3). Distribution of roosts among decay stages differed pre- and post-treatment within the primary removal colony but not in the control colony or the secondary removal colony (Table 3). Distribution of roosts among crown classes differed pre- and post-treatment for the primary removal colony but not in the control or secondary removal colony (Table 3). Distribution of roosts among decay stage and crown classes did differ among colonies in some cases (Table 3). We found no difference in roost species composition between pre- and post-treatment or among any of our groups (Table 3). Sassafras (*Sassafras albidum*) trees or snags were the most commonly used roost species, accounting for between 43 and 57% of roosts used in each group.

**Table 3:** Summary of female northern long-eared bat roost characteristics.

	**Control**		**Primary Roost Removal**		**Secondary Roost Removal**	
	**Pre**	**Post**	**Pre**	**Post**	**Pre**	**Post**
dbh (cm)	31.6 (± 4.6)	32.2 (± 15.0)	34.6 (± 22.2)	34.5 (± 14.5)	30.5 (± 24.5)	30.8 (± 16.4)
Height (m)	13.4 (± 9.5)	18.0 (± 8.3)	15.4 (± 8.3)	17.7 (± 9.1)	14.7 (± 7.1)	15.4 (± 8.0)
Canopy Openness (%)	5.7 (± 4.1)	4.1 (± 2.9) ^a^	4.7 (± 4.6) ^b^	5.4 (± 3.4) ^c,d^	4.1 (± 8.2) ^a,c^	2.0 (± 3.2) ^b,d^
Decay Stage (% in stage)	^a^	^b,c^	^b,d^	^a,d,e^	^a,e^	
Stage 1	0.0	17.4	15.2	35.7	9.5	17.1
Stage 2	50.0	21.7	12.1	23.8	28.6	14.3
Stage 3	0.0	21.7	12.1	14.3	19.0	17.1
Stage 4	0.0	13.0	18.2	19.0	9.5	37.1
Stage 5	25.0	17.4	18.2	4.8	28.6	11.4
Stage 6	25.0	8.7	24.2	2.4	4.8	2.9
Crown Class (% in class)	^a^	^b^	^b,c^	^a,c,d^	^d^	-
Suppressed	75.0	17.4	69.7	7.1	66.7	34.3
Intermediate	25.0	47.8	15.2	57.1	9.5	40.0
Co-dominant	0.0	21.7	6.1	26.2	9.5	14.3
Dominant	0.0	13.0	9.1	9.5	14.3	11.4

Summary of roost characteristics (mean ± SD) for 3 northern long-eared bat (*Myotis septentrionalis*) maternity colonies subjected to different levels of roost removal on the Fort Knox military reservation, Kentucky, USA, pre- and post- roost removal (2011 and 2012) treatment. Significant differences (*P* < 0.05) between groups are indicated by superscripts a-e.

**Figure 1:**
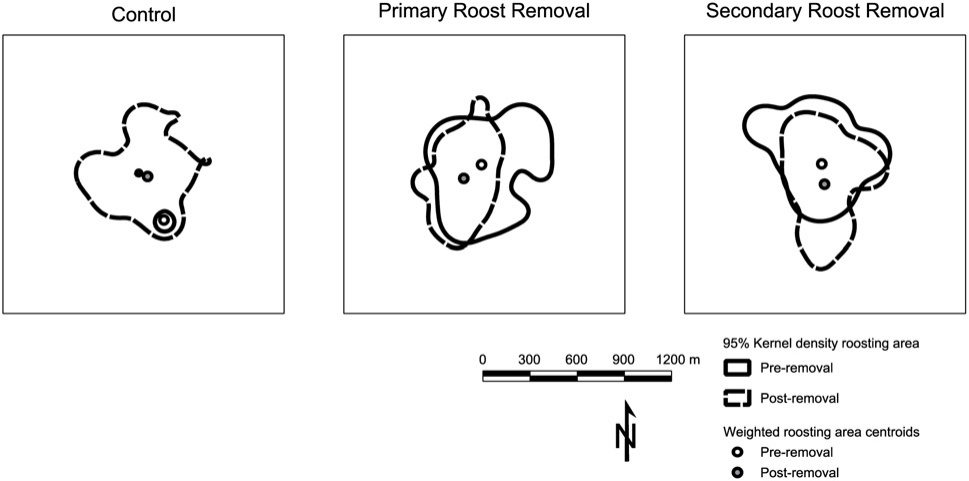
Northern long-eared bat maternity colony roosting areas. Roosting areas (95% utilization distribution) of 3 northern long-eared bat (*Myotis septentrionalis*) maternity colonies subjected to different levels of roost removal on the Fort Knox military reservation, Kentucky, USA, pre- and post- roost removal (2011 and 2012)

Distances moved between sequentially used roosts were non-normally distributed with right skew; median distances were between 111.1 and 219.4 m (Table 1). Distances between sequentially used roosts differed only pre- and post-roost removal in our secondary roost removal treatment group (model R^2c^ = 0.18, R^2m^ = 0.08; Tables 1, 4). Overall colony roosting areas were between 1.3 and 58.5 ha (Table 1). Patterns of roosting area space use largely were consistent between pre- and post-treatment in our primary and secondary roost removal treatment groups, particularly evident in the distances between weighted colony roosting area centroids (Table 1, Fig. 1). However, space use by and roosting area centroids of our control colony differed substantially between years (Table 1).

**Table 4:** Factors influencing distances moved between roosts by female northern long-eared bats.

**Predictor**	**Parameter Estimate**	**SE**	**t value**	***P*-value**
Intercept	4.50	0.50	0.503	< 0.001
Post-removal	0.47	0.52	0.520	0.37
Treatment: Primary	0.41	0.52	0.519	0.43
Treatment: Secondary	-0.23	0.55	0.547	0.68
Repro: Non-reproductive	0.79	0.43	0.433	0.07
Repro: Post-lactation	-0.17	0.22	0.217	0.44
Repro: Gestating	0.53	0.23	0.227	0.02
Post-removal x Primary	-0.36	0.55	0.549	0.52
Post-removal x Secondary	0.46	0.58	0.580	0.43

Parameter summary of the linear mixed model describing the log transformed non-zero distances moved between sequentially used roosts by female *Myotis septentrionalis* in 3 maternity colonies subjected to different levels of roost removal on the Fort Knox military reservation, Kentucky, USA, pre- and post- roost removal (2011 and 2012) treatment. Repro = bat reproductive condition.

Roost network degree centralization significantly was greater than random for primary removal and control colonies, but not the secondary roost removal colony pre-treatment (Table 1). Roost network clustering differed from random networks in both the primary and secondary roost removal colonies post-treatment, but, for all other colonies, there was no difference from random networks (Table 1). Roost network density did not significantly differ from random networks for any group (Table 1). As represented in the social networks, bats shared between 3.5 and 15.9 social connections with other bats within colonies (Table 5). Social network degree centralization differed from random networks only for the control colony pre-treatment and the primary roost removal treatment post-treatment; the former was significantly less than and the latter significantly greater than equivalent random networks (Table 5). Social network clustering significantly was greater than that of random networks for colonies except the secondary roost removal treatment colony pre-treatment (Table 5). Social network density did not differ from random networks pre-treatment, but was greater in all other cases (Table 5).

**Table 5:** Northern long-eared bat maternity colony social network metrics.

	**Control**		**Primary Roost Removal**		**Secondary Roost Removal**	
	**Pre**	**Post**	**Pre**	**Post**	**Pre**	**Post**
Minimum Colony Size	18	20	14	25	13	24
Number of Bats Tracked	15	14	13	25	8	23
Mean Bat Degree	14.0 (± 0.0)	6.7 (± 2.7)	4.6 (± 2.6)	15.9 (± 5.3)	3.5 (± 1.9)	6.1 (± 2.1)
Network Degree Centralization	0 (<)	0.38	0.33	0.37 (>)	0.48	0.14
Network Clustering Coefficient	1 (>)	0.76 (>)	0.74 (>)	0.93 (>)	0.64	0.77 (>)
Network Density	1	0.51	0.38	0.66	0.5	0.28

Pre- and post- roost removal (2011 and 2012) treatment social network metrics for 3 northern long-eared bat (*Myotis septentrionalis*) maternity colonies subjected to different levels of roost removal on the Fort Knox military reservation, Kentucky, USA. Where appropriate, when network metrics differed from random networks, the arrow indicates the direction of difference.

**Figure 2:**
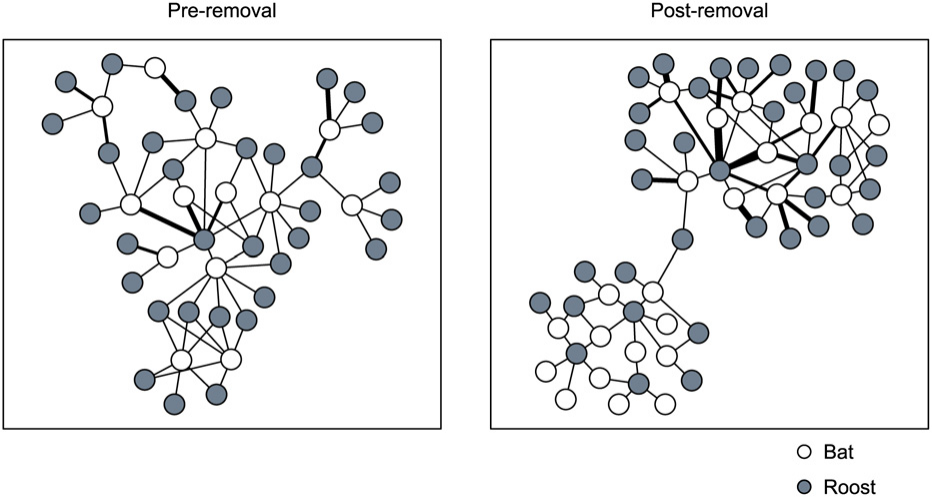
Northern long-eared bat maternity colony roost network map. Pre- and post- roost removal treatment (2011 and 2012) 2-mode roost network map of a northern long-eared bat (*Myotis septentrionalis*) maternity colony subjected to removal of 5 secondary roosts on the Fort Knox military reservation, Kentucky, USA. Edge width is scaled by the number of connections between a bat and an individual roost.

Visual inspection of the roost network maps indicated that the secondary roost removal colony was split into 2 groups connected only by a single roost post-treatment (Fig. 2). Because these 2 halves possibly represented 2 separate colonies connected by a single ‘chance’ roost use, we conducted a *post-hoc* analysis wherein we removed the roost connecting the 2 network sections (subcolony 1 and subcolony 2) and re-calculated spatial metrics. Roosting area was 46.37 ha for subcolony 1 and 27.43 ha for subcolony 2. Roosting areas of these 2 sections overlapped substantially (UDOI = 1.26).

## Discussion

In our manipulative roost removal experiment, treatments did not result in abandonment of roosting areas by northern long-eared bats. Persistence after exclusion from a roost also has been observed in big brown bats (*Eptesicus fuscus*) in northern forest-prairie transitions zones in Canada [15] and disc-winged bats (*Thyroptera tricolor*) in Costa Rican tropical forests [18], species that both exhibit relatively frequent roost switching. In contrast, syntopic little brown bats (*Myotis lucifugus*), that form larger colonies and roost-switch less than northern long-eared bats, appear to abandon roosting areas after exclusion [16]. Persistence after roost loss may be related to the greater number of roosts used by colonies and to roost ephemerality. Roost fidelity is less in species with more ephemeral roosts [74], therefore, having a variety of alternate roosts or some degree of flexibility in what roosts may be selected may be an adaptation for tolerating roost loss for the northern long-eared bat.

Northern long-eared bat maternity colony roosting areas did not appear to change as a result of either of our roost removal treatments. In contrast, Chaverri and Kunz [18] found that exclusion resulted in larger individual roosting home ranges in disc-winged bats [18] and Borkin et al. [17] found that roost loss resulted in smaller home ranges in New Zealand long-tailed bats (*Chalinolobus tuberculatus*) [17]. Increased home range size in disc-winged bats was related to the need to locate a limiting resource—suitable roosts [18]. However, northern long-eared bats are not extreme roost specialists [32, 75, 76] and potential roosts are not limited on our sites [77]. On the other hand, decreased home range size in New Zealand long-tailed bats as a result of roost loss following clear-cutting, reflected the lack of available roosts and alternative roosting areas in the harvested areas [17]. Locally, large numbers of available roosts may explain why so few roosts were used in both years of our study and why colony locations did not change.

It was surprising that so few roosts were used both pre- and post-treatment, but could be the result of tracking different bats in each year. We captured a substantial proportion of the bats within individual colonies (range 0.62–1.0, $\bar{x}=0.84$). As such, it is unlikely that our low recapture rate was due to sampling effort. Regardless, roost removal treatments did not impact the number of roosts used by individual bats within treatment areas when controlling for the number of total locations and reproductive condition. The lack of difference in the number of roosts used differs from Borkin *et al.* [17], who found that bats used fewer roosts post-roost loss. The number of roosts used per bat was fewer in 2011 than in 2012 in our control colony, but this is likely due to the fact that the colony was captured and tracked during parturition in 2011 [78]; the number of roosts used per bat in the control colony in 2012 was consistent with that of all other groups. Given the positive relationship between the number of roosts located and the number of days a bat was tracked, differences in the total number of roosts located per colony were not unexpected.

Northern long-eared bats are known to exhibit inter-annual site fidelity of at least 5 years in a mixed pine-deciduous system in Arkansas [79], but our low recapture rates relative to our sampling effort suggest that bats marked during the first year of our study largely were not present in the second. Whether this is due to high annual adult mortality or some other socio-spatial assortment dynamic is unknown, but Perry [79] also recaptured few banded individuals. Consistent patterns of space use between years suggest that, although colony composition changed, colony identity did not. Northern long-eared bat maternity colonies [80] as well as those of some other species [81] contain maternally-related individuals, and it is possible that primarily juveniles from the first year returned in the second. In the context of having tracked different bats within colonies, our data may be interpreted best not as changes in behavior of individual bats resulting from removal treatments, but as differences in patterns of colony behavior at our treatment sites.

In contrast to Chaverri and Kunz [18], we observed no change in roost species selection post-roost removal. This is consistent with the high roost availability at our sites [27]. Roost decay stage and crown class in the primary removal colony were the only roost characteristics to differ between pre- and post-treatment. Selection for more advanced stages of decay in 2011 appears to be correlated with crown class, as trees in advanced stages of decay at our sites are primarily in suppressed crown classes. Although the difference in decay stage and crown class pre- and post-treatment is statistically significant only for the primary removal colony, a similar trend in reduced selection for suppressed roosts in later stages of decay was visible across all colonies in 2012. It is possible that by random chance roost removal caused the difference in roost decay stage and crown class in our findings, but given the lack of difference between roost dbh, height, and canopy openness in the primary removal colony, this seems unlikely. Higher summer temperatures in 2011 than in 2012 on our study site may have caused bats to select trees in more suppressed crown classes, thereby reducing solar heating of roosts. Mean minimum temperature during June–July was 1.78 C° greater in 2011 than in 2012 (National Oceanic and Atmospheric Administration station GHCND: USC00154955); similarly small temperature differences have been found to affect roost selection by Bechstein’s bats (*Myotis bechsteinii*) [82] and development of juvenile greater mouse-eared bats (*Myotis myotis*) [83].

Patterns of northern long-eared bat roost use and association, as assessed through roost and social networks, displayed a mix of random and non-random characteristics. The overall character of roost networks relative to random networks was similar within and among treatments. Although there were minor differences in roost and social networks pre- and post-treatment, northern long-eared bat social network structure changes with reproductive condition [84, 85]. After accounting for reproductive condition, the character of the roost networks post-treatment differed only for roost network clustering. The change in roost network clustering from not significantly different from random networks to significantly greater than random networks also was reflected through increased social network density. An increase in roost network clustering and social network density may be an adaptive response to maintain colony stability after roost loss. Such an adaptive response to roost loss could suggest co-evolution between northern long-eared bats and these mixed mesophytic forests and other systems with similar stand dynamics and disturbance patterns, but replication of our study across more regions and forest types is required to document this.

For the secondary roost removal colony, we observed a segmented roost network and the only statistically significant difference in the distance moved between sequentially used roosts. Division of this network into 2 halves as a result of the removal of 24% of roosts would be consistent with previous simulation based outcomes showing that loss of approximately 20% of roosts generates a 50% chance of colony fragmentation [27]. Connection of the 2 halves of this network by a single roost may reflect an incomplete division of the colony. An incomplete division may indicate that colony fragmentation occurs incrementally as roosts are lost, an outcome that theoretically should be most likely to occur if individual roosts are important locations for social interaction. Incomplete colony fragmentation is consistent with our finding that the 2 sections of this colony shared a single roosting area—an observation that was contrary to our *a priori* prediction that colony fragmentation would result in random use of the roosting area, but that may be related to the difference in distances moved between roosts by bats in this colony. Alternately, apparent division also could be the result of unwarranted joining of two separate neighboring colonies as a result of chance use of single roost. Silvis *et al.* [27] speculated that roost sharing may be infrequent and inconsequential at the periphery of the roosting area for northern long-eared bats. In this case, the shared roost was not at the periphery of the colony roosting area and the roosting areas of the 2 sections of the colony overlapped extensively in terms of both extent and concentration of use. Research from other bat species in both temperate and tropical regions suggests that roosting areas are exclusive relatively to individual colonies [17, 30, 31]. Whether this apparent fragmentation is a result of roost removal treatments or some other process remains speculative.

## Conclusions

In their review of conservation concerns for bats in the United States, Weller et al. [86] identified a need to transition conservation priorities from focal threats to diffuse threats. In the context of the White-nose Syndrome enzootic that is threatening many species, including the northern long-eared bat, with widespread extirpation, it is necessary to link focal and diffuse threats through understanding of the impacts of specific changes to roosting habitats. Although our study contains limited replicates of our individual treatments, it is to our knowledge the only study to perform targeted roost removal treatments for colonial bats in a temperate forest ecosystem. Clearly, caution should be taken in interpreting the results of individual treatments, particularly with regard to changes in roost and social network structure. However, our results are consistent with previous predictions and anecdotal observations that northern long-eared bats would be robust to low levels of roost loss [20, 22] particularly if loss of these naturally ephemeral roost resources are lost at or below rates of tree mortality / snag loss in temperate forests. Clearly, the maximum levels of annual or cumulative multi-year roost loss that northern long-eared bats can tolerate remains to be determined. It is important to consider that roosts were not limiting at our study sites similar to much of the temperate forested environments where northern long-eared bats occur [10, 87]. However, in more roost limited areas, e.g., in agricultural landscapes with greater forest fragmentation or in industrial forest settings skewed towards younger forest age classes, roost loss may have different consequences for northern long-eared bats.

Monitoring of sufficient numbers of colonies for robust inference is largely infeasible within a single study. Therefore, replication across studies is needed to better confirm or modify the patterns we have observed. With the ongoing spread of White-nose Syndrome in North America, and continued rapid declines in northern long-eared bat populations, replication of this study in disease-free areas is urgently needed. Moreover, a better understanding the impacts of roost loss, whether natural or anthropogenic, on survival and recruitment remains a critical gap in our knowledge of bat ecology.
